# Mapping Evidence of Self-Sampling to Diagnose Sexually Transmitted Infections in Women: A Scoping Review

**DOI:** 10.3390/diagnostics12081803

**Published:** 2022-07-26

**Authors:** Ziningi N. Jaya, Witness Mapanga, Brian van Niekerk, Thobeka Dlangalala, Kabelo Kgarosi, Mathias Dzobo, Delarise Mulqueeny, Tivani P. Mashamba-Thompson

**Affiliations:** 1 School of Health Systems and Public Health, Faculty of Health Sciences, University of Pretoria, Pretoria 0002, South Africa; u15252061@up.ac.za (W.M.); u10225120@tuks.co.za (T.D.); mattdzb@gmail.com (M.D.); 2 Department of Biomedical Science, Faculty of Natural Science, Mangosuthu University of Technology, Umlazi 4031, South Africa; 3 Department of Plant and Soil Sciences, Faculty of Natural and Agricultural Sciences, University of Pretoria, Pretoria 0002, South Africa; u19012544@tuks.co.za; 4 Department of Library Services, Faculty of Health Sciences, University of Pretoria, Pretoria 0002, South Africa; kabelo.kgarosi@up.ac.za; 5 Department of Social Work, Faculty of Arts, University of Zululand, Richards Bay 3900, South Africa; mulqueenyd@unizulu.ac.za; 6 Faculty of Health Sciences, University of Pretoria, Pretoria 0002, South Africa; tivani.mashamba-thompson@up.ac.za

**Keywords:** self-sample, sexually transmitted disease, women

## Abstract

Background: Sexually transmitted infections (STIs) are a major global healthcare burden, disproportionately affecting women. Self-sampling interventions for diagnostic purposes have the potential to improve STI healthcare management and expand STI services. However, there is currently no published evidence of the global use of self-sampling interventions to diagnose STIs in women. The main aim of this scoping review was to map evidence on the use of self-sampling interventions to diagnose STIs in women. Methodology: The methodology of this scoping review was guided by Arksey and O’Malley and Levac. A comprehensive literature search was conducted in PubMed, Scopus, Web of Science, Medline (EBSCO), ProQuest, and Cochrane. For grey literature, a search was conducted in Open Grey, World Health Organization, Google, and conference proceedings and dissertations. All search results were screened and assessed for eligibility. Thereafter data from eligible studies was extracted and analysed. The quality of these studies was appraised using the Mixed Methods Appraisal Tool 2018 version. Results: A total of 770 articles were retrieved from databases and grey literature sources. A total of 44 studies were eligible for data extraction following title, abstract and full-text screening. Of the included studies, 63% presented evidence of research conducted in high-income countries and 37% presented evidence in low- and middle-income countries. Studies presented evidence on the following: feasibility of self-sampling in remote areas; acceptance and ease of use of self-sampling interventions; types of self-sampled specimens; pooled samples for diagnosing STIs; laboratory diagnostic assays for STI using self-sampled specimens; and self-testing of self-sampled specimens. Conclusions: Self-sampling interventions are feasible and easy to use and, therefore, can improve STI management and treatment in women across various age groups and various access levels to good-quality healthcare. Despite this, there is a lack of evidence of self-sampling interventions designed according to user preferences. We recommend studies to collaborate with women to co-develop user-friendly self-sampling interventions to diagnose STIs in women.

## 1. Introduction

Sexually transmitted infections (STIs) are a global health challenge, with one million new cases diagnosed every day [1]. Although STIs affect both genders, women are at a higher risk due to the anatomy of their reproductive tract [2]. STIs are commonly diagnosed and treated based on the presentation of symptoms, particularly in low- and middle-income countries (LIMC) where access to technologically advanced diagnostic procedures are limited [3]. Often, STIs are treated using a syndromic management approach, where the patient is treated for a group of conditions that cause similar symptoms and often occur concomitantly. Although treating symptomatic STIs is effective, many asymptomatic infections are missed [4]. Not diagnosing or treating asymptomatic STIs may result in infections persisting or spreading. Diagnosing STIs mostly requires physically examining people who present to healthcare facilities [3], which may be challenging in remote areas where access to healthcare is limited [5,6]. Physical exams are unattractive to many people, due to the invasive nature of physical exam procedures and the social stigma associated with STIs [5,6]. Delayed diagnosis and treatment of STIs often increase the risk of STI-related long-term health complications, including chronic pelvic pain, fertility issues, and cervical cancer development [7].

Self-sampling to diagnose STIs is widely used in high-income countries (HIC) as an alternative to having healthcare workers collect samples [8]. Through self-sampling, people can collect their specimens, either at healthcare facilities or at home, in relative privacy [7,8]. Allowing people to self-sample at their convenience eliminates various barriers often associated with STIs, such as lack of privacy and stigmatization [7,9]. Self-sampling may also promote the diagnosis and management of STIs in remote areas and allow people who are skeptical and uncomfortable with conventional clinic-based practices to access treatment [5]. Self-sampling is also effective in screening for asymptomatic infections [6,7]. As a means of scaling up global STI services, the World Health Organization (WHO) recommends the expansion of self-sampling [10]. Despite this recommendation, self-sampling interventions to diagnose STIs in women are not very well documented.

The long-term effects of undiagnosed and untreated STIs, together with the difficulties associated with clinic-based management of STIs, contributes to the global challenges associated with STI management [11,12]. Self-sampling has the potential to facilitate STI management and expand STI services. The aim of this scoping review is to map evidence on the use of self-sampling interventions to diagnose STIs among women. Our findings may assist policymakers and healthcare practitioners involved in sexual healthcare and inform future research on self-sampling interventions for diagnosing STIs in women.

## 2. Materials and Methods

This scoping review was part of a larger study aiming to develop a user-friendly self-sampling intervention to diagnose STIs among young women in poor urban communities in eThekwini District Municipality, in KwaZulu-Natal, in South Africa. The scoping review was guided by recommendations from Arksey and O’Malley [13], Colquhoun Levac [14], and Godfrey Peters [15]. We present our methods and findings using the preferred reporting items for systematic reviews and meta-analyses extension for scoping reviews (PRISMA-ScR) guideline [16]. The scoping review protocol was registered prospectively on Open Science Framework and can be accessed via the link: https://osf.io/tnbx6 (accessed on 20 June 2022).

### 2.1. Identifying the Research Question

We asked the research question: What is the evidence on self-sampling interventions to diagnose STIs among women?

We adopted the population, concept, and context (PCC) framework to effectively address the research question (see Table 1).

### 2.2. Identifying Relevant Studies

We conducted a systematic literature search of the following databases: PubMed, Scopus, Web of Science, Medline (EBSCO), ProQuest, and Cochrane. We used medical subject headings (MeSH) terms to define our searches with Boolean operators (AND/OR) between search terms. The search terms included (1) “self-sample” or “self-collect” or “self-administer” or “self-obtain”, (2) “sexually transmitted infections”, (3) “diagnostic specimens” or “diagnostic samples”, and (4) “women”. We searched the grey literature on the following websites: Open Grey, WHO, Google, and conference proceedings and dissertations. We adjusted keywords to suit different databases. We did not apply any time or language restrictions to ensure that we captured most of the literature. An experienced librarian conducted comprehensive database searches to ensure that the best search strategies were used for each database.

We included articles that fulfilled the following criteria:

- Peer-reviewed journal articles;
- Studies presenting evidence on self-sampling interventions for STIs;
- Studies presenting evidence on self-sampling in women for STI diagnosis;
- Studies of all designs with relevant information; and
- Studies focussing on the type, acceptability, feasibility, and effectiveness of self-sampling.

Articles were excluded if they:

- Focused on self-sampling interventions for HIV only; and
- Only presented evidence of specimens collected by healthcare workers for STI diagnosis.

### 2.3. Selection of Studies

Studies were selected in three stages. Firstly, article titles were screened according to their title in line with eligibility criteria. Eligible articles were exported to reference-manager software. In the second phase, two independent reviewers screened abstracts, using a screening tool that outlined the eligibility criteria. The screening tool was calibrated to ensure the accuracy and utility of screening questions. Calibration involved randomly selecting 21 (10%) articles from 211 articles, and then, pilot screening using the screening tool. The reviewers held extensive discussions to resolve any discrepancies and amend the screening tool accordingly. After the second stage of screening, eligible publications were exported to reference-manager software. The third stage included screening full texts using the screening tool. A third reviewer helped to resolve any discrepancies arising from full-text screening. Kappa statistics were used to determine the level of agreement between screeners.

### 2.4. Data Charting

We developed a data charting tool with variables relevant to the research question. Two independent reviewers then piloted the data-extraction tool, using seven (10%) of the included studies. The reviewers discussed the results of the extraction tool and updated the tool accordingly. Data were extracted from each article and thematically organised in a spreadsheet. Extracted data included: author, aim, study design, country, study population and sample size, type of self-collected specimen, diagnostic test used, key findings and conclusions.

### 2.5. Quality Appraisal of Included Articles

Included articles were critically appraised using the Mixed Method Appraisal Tool (MMAT), version 2018 [17]. Included articles were grouped according to study design, either qualitative or quantitative, and appraised using the relevant sections of the MMAT. Articles were scored as follows—low-quality studies had MMAT scores below 50%, average-quality articles had MMAT scores between 51–75%, and high-quality articles had MMAT scores ranging from 76–100%.

### 2.6. Collating, Summarising, and Reporting Results

The included articles were thematically analysed to demonstrate how they related to the research question. The following themes emerged from the included articles: feasibility, acceptance and ease of self-sampling interventions; types of self-collected specimens; diagnostic accuracy of self-collected specimens; agreement between physician-collected specimens and self-sampled specimens; pooled specimens for STI diagnosis; and self-testing of self-collected specimens. Our research findings were narratively summarised.

## 3. Results

### 3.1. Screening Results

**Figure 1:**
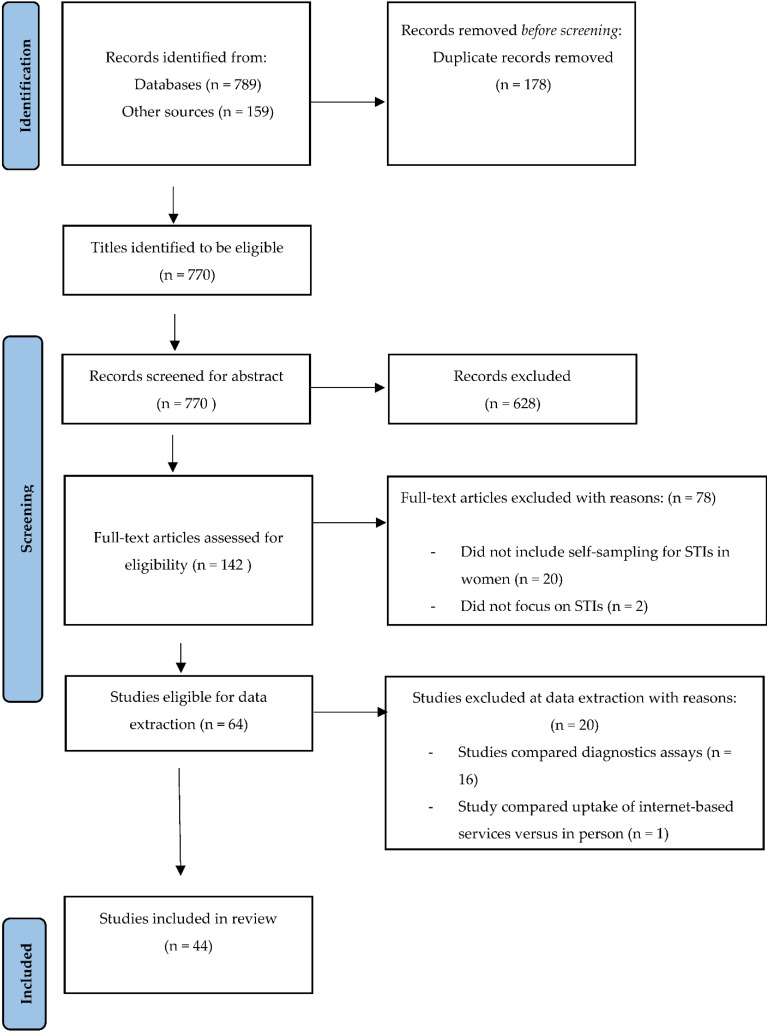
Preferred Reporting Items for Systematic Reviews and Meta-Analyses (PRISMA) flow diagram of the study selection process.

Our search and screening strategy is outlined in the PRISMA flow diagram (Figure 1). We retrieved and screened 770 articles during title screening, of which 681 were from databases, nine were from Google, and 80 were from the WHO website. Databases search results are contained in Table 2. This was followed by abstract screening, after which 628 articles were excluded. We screened the full texts of the remaining 142 studies, of which 78 were excluded. At this stage, articles were excluded because they did not include self-sampling for STIs in women (*n* = 20), they did not focus on STIs (*n* = 2), and they did not assess the accuracy or validity of results of self-collected specimens (*n* = 56). The remaining 64 studies were eligible for data extraction. During data extraction, we excluded 20 articles because they compared laboratory diagnostic assays (*n* = 16), compared uptake of internet-based services versus in-person services (*n* = 1), and did not focus on STIs (*n* = 1) or on self-sampling in women (*n* = 2). Ultimately, 44 studies were included for review.

Reviewers showed moderate agreement following full-text screening (ƙ = 0.82, *p* < 0.05). McNemar’s chi-square statistic suggested that reviewers had similar proportions of yes/no answers (*p* > 0.05).

### 3.2. Quality Appraisal

Of the 44 studies included in review, 36 studies were primary studies. The quality of these studies was appraised using the MMAT 2018 version [17]. The overall score of the studies ranged between 65% and 100%. Nine studies had an average score of 60–75% [18,19,20,21,22,23,24,25,26] and seven other studies scored an average score of 65%. The remaining 27 studies scored a high-quality score between 76–100% [6,27,28,29,30,31,32,33,34,35,36,37,38,39,40,41,42,43,44,45,46,47,48,49,50,51,52,53].

### 3.3. Characteristics of Studies

**Figure 2:**
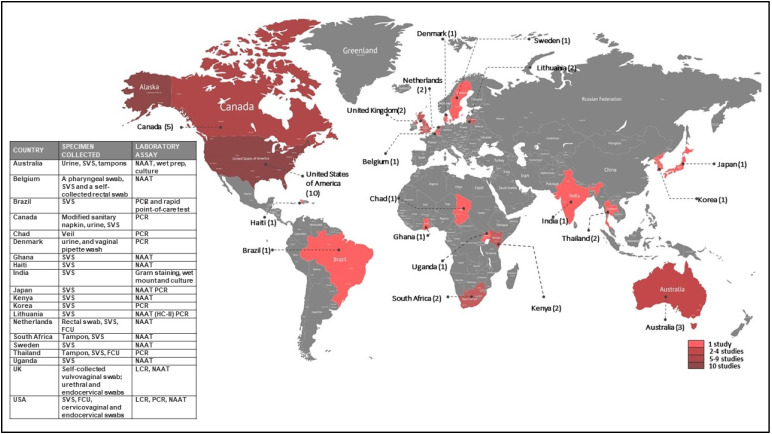
World map showing global evidence on self-sampling interventions for diagnosing STIs in women.

The characteristics of the 44 included studies are summarised in Table 3. Studies were conducted in various HICs and LMICs (Figure 2). Eleven (24%) studies were conducted in the United States of America (USA) [20,22,28,29,33,35,37,45,47,54,55], five (11%) in Canada [21,52,53,56,57], three (7%) in Australia [32,41,58], two (5%) in the United Kingdom (UK) [25,38], and two (4%) in The Netherlands [44,59]. Two studies (4%) were conducted in South Africa [27,60], two (4%) in Lithuania [40,48], and two (4%) in Kenya [23,30]. Only one (2%) study was conducted in each of the following countries: Brazil [60], Sweden [46], Korea [42], Ghana [36], Japan [31], Uganda [34], Haiti [51], Thailand [49], Belgium [26], Denmark [24], India [43], and Chad [6]. In addition, four (8%) studies were systematic reviews and meta-analyses and were not assigned any specific study location [18,19,39,61].

Nucleic acid amplification-based tests (NAATs) were used to diagnose STIs in 95% (*n* = 42) of studies [6,18,19,20,21,23,24,25,26,27,28,29,30,31,32,33,34,35,36,37,38,39,40,42,44,45,47,48,49,51,52,53,54,55,57,58,59,60,62,63,64], while only one study used a NAAT, conventional culture, and wet mount techniques [41]. The NAAT tests included Aptima Combo 2, Polymerase Chain Reaction (PCR), IVD-marked multiplex real-time PCR Allplex STI Essential Assay, Digene Hybrid Capture II (HCII) *Chlamydia trachomatis/ Neisseria gonorrhoea* (CT/NG) Test, PCR for CT (cobas^®^ TaqMan 48 real time PCR), cobas^®^ Amplicor CT/NG test, the Anyplex II Huma papillomavirus (HPV)28 Detection assay, Real-time PCR using CFX9, care HPV Assay, Ligase Chain Reaction (LCR), and Strand Displacement Amplification. One study used only conventional wet mount and culture techniques to diagnose STI [43]. Only one study used NAAT and point-of-care (POC) devices to detect infection [61].

### 3.4. Summary of Findings

We reviewed studies that presented evidence on using self-sampled specimens for diagnosing STIs in women across the globe. The following themes emerged from the included studies: feasibility, acceptance and ease of self-sampling interventions; types of self-sampling specimens; diagnostic accuracy of self-sampled specimens; agreement between physician-collected specimens and self-sampled specimens; pooled samples for STI diagnosis; and self-testing of STIs using self-collected specimens.

#### 3.4.1. Feasibility, Acceptance, and Ease of Self-Sampling Interventions

Nine studies reported on acceptance, ease of use, and feasibility of self-sampled specimens in settings where pelvic examinations were not routinely conducted and healthcare access was limited [23,27,33,38,42,51,52,54,56]. In Haiti, Boggan et al. [51] reported good feasibility of self-sampling for cervical cancer screening. Similarly, Korean women also found that self-sampled vaginal swabs were feasible for detecting HPV DNA and cervical cancer screening [42]. In South Africa, some women preferred pelvic examinations conducted by attending healthcare workers, even though self-sampling was feasible and acceptable [27]. In contrast, Arias et al. [52] and Morris and Rose [18] found than women preferred self-sampling and avoided pelvic examination by healthcare workers. Similarly, women in the USA [54], Canada [56], Kenya [23], and the UK [38] reported that self-sampling was easy. Although most women preferred self-sampling, there is relatively limited evidence for interventions tailored to patients’ preferences, in terms of specimen type, place of specimen collection, communication of results, and management and treatment of infected individuals.

#### 3.4.2. Types of Self-Sampled Specimens

Studies in the review investigated the use of different types of self-collected specimens to diagnose various STIs, including NG, CT, TV, HPV and genital mycoplasmas. Self-sampled specimens were collected using vaginal swabs, cervicovaginal swabs, rectal swabs, pharyngeal swabs, urine and tampons. Thirty-three studies used vaginal swabs [18,19,21,22,25,26,27,28,29,31,33,34,35,36,38,39,40,41,42,43,44,45,46,48,49,51,52,54,55,56,57,59,60,61], four studies used cervicovaginal swabs [23,30,47,61], 12 studies used urine specimens [22,24,28,33,38,39,41,45,46,51,53,58,59], nine studies used tampons [18,20,27,32,37,41,49,51,61], three studies used rectal swabs [26,39,44], two studies used pharyngeal swab [26,39], one study used a modified sanitary towel [53], one study used a vaginal wash specimen [24], and one study collected genital specimens using a veil collection device [6]. Of the 33 studies that collected vaginal swabs, 18 studies used multiple types of self-sampled specimens including vaginal swabs, rectal swabs, pharyngeal swabs, tampons, and urine.

Two studies in the USA [33,35] and one study in Japan [31] concluded that self-sampled vaginal swabs were accurate and suitable for diagnosing STIs. Self-sampled vaginal swabs also showed high sensitivity and specificity in Brazil and South Africa [60], Canada [53], the USA [45], Japan [31], and the UK [38].

In the USA, Fang et al. [45] demonstrated that urine was the least sensitive method for diagnosing STIs. In Australia, urine specimens transported from remote settings were least sensitive [41]. In the Netherlands, STIs were similarly detected by self-sampled vaginal swabs and by a combination of vaginal swabs and first-catch urine [59]. Levy et al. [39] reported that urine was the preferred self-sampling specimen type for men.

Self-sampling was also conducted using tampons. In the USA, tampons were highlighted as a sampling technique that could collect a bigger cell sample than vaginal swabs and, therefore, had the potential to rapidly diagnose women [20]. Chandeying et al. [49] in Thailand reported that tampons were sensitive in detecting infections. However, another study conducted in USA, indicated that a high proportion of tampons were insufficient for STI testing [37].

Two Kenyan studies [23,30], one USA study [47], and a meta-analysis by Ogilvie et al. [61] investigated the use of self-sampled cervicovaginal swabs. In these studies, self-collected cervicovaginal swabs were deemed acceptable and valid for self-sampling even in places where pelvic examinations are not done routinely [23,47,48]. In Chad, one study investigated the use of a specimen collection device called a veil, which was reported as a convenient and gentle way to collect cervicovaginal secretions for STI testing [6].

#### 3.4.3. Diagnostic Accuracy in Self-Collected Specimens

Of the 44 included studies, 25 studies reported on the accuracy of laboratory diagnostic results [6,18,19,22,25,31,34,35,36,37,38,41,42,43,44,45,48,49,51,53,55,58,60,61]. In Canada, Alary et al. [53] reported that a self-collected modified sanitary towel had a sensitivity and specificity of 93.1%. In Thailand, Chandeying et al. [49] reported diagnostic accuracy of tampons (sensitivity = 95.9%, specificity = 98.4%), urine (sensitivity = 70.3%, specificity = 99.7%), endocervical swabs (sensitivity = 59.5%, specificity = 99.7%), and vaginal swabs (sensitivity = 89.2%, specificity = 99.2%). In Lithuania, Domeika et al. [48] reported that vaginal swabs had 100% sensitivity and specificity, when analysed with a PCR assay. Similarly, Jang et al. [19] reported that vaginal swabs had a sensitivity and specificity of 97.2% and 97.6% respectively. Geelen et al. [44] also reported that rectal swabs and vaginal swabs had a sensitivity and specificity of 87.1% and 100%, respectively. Irrespective of self-sample type, our findings highlight that diagnostic testing on self-collected specimens yields fairly accurate results.

#### 3.4.4. Agreement between Physician-Collected and Self-Sampled Specimens

We reviewed 19 studies that compared physician-collected and self-sampled specimens [6,19,21,23,28,29,30,31,34,36,37,39,43,45,47,51,55,56,57,59]. Boggan et al. [51] reported 91.4% agreement between self-sampled vaginal swabs and physician-collected cervical specimens. In Canada, Chernesky et al. [21] reported 82% agreement between self-collected vaginal swabs and physician-collected cervical specimens. De Marais et al. [47] reported strong agreement between self-samples collected at home and in the clinic, and between self-samples collected at home and physician-collected specimens. According to Boggan et al., [51] the strong agreement between vaginal swabs and cervical specimens suggests that self-sampled vaginal swabs could be used to improve access to STI healthcare services in high-risk populations.

#### 3.4.5. Pooled Specimens for STI Diagnosis

Two studies explored the use of pooled specimens to diagnose STIs [26,65]. In both instances, pooled specimens reportedly saved costs, and enabled more patients to be tested which increased the rate of STI detection [26,65]. Pooling samples may thus be useful for detecting STIs. Our review reveals a large knowledge gap on the use of pooled patient specimens to diagnose STIs.

#### 3.4.6. Self-Testing of Self-Collected Specimens

Only one USA study reported on the use of self-testing assays on self-collected samples [54]. This study describes self-testing of STIs using self-collected specimens in adolescent females [54]. Young women found self-testing and self-sampling to be acceptable, more so than having to undergo a pelvic exam [54]. These findings highlight the need for innovative and convenient diagnostic tools to diagnose STIs beyond healthcare to improve STI treatment and management services.

## 4. Discussion

This scoping review presents global evidence on self-sampling interventions used to diagnose STIs in women. Our findings show that 23% of included studies were conducted in the USA and 95% (*n* = 42) of the included studies used NAAT to diagnose or detect STIs. We found few studies describing participant-tailored self-sampling interventions that could be used for routine STI management at local healthcare facilities. Most studies investigated the use of self-sampled vaginal swabs to diagnose STIs [18,19,21,25,26,27,28,29,31,33,34,35,36,38,39,40,41,42,43,44,45,46,48,49,51,52,54,55,56,57,59,60,61] compared to urine, tampons, and sanitary napkins, similar to results reported elsewhere [62]. We also found limited evidence of testing self-collected specimens using rapid near-patient diagnostic assays for diagnosing STIs. The WHO World Health Day 2019 Campaign Essentials [66] emphasizes the drive for universal health coverage through primary healthcare services. All people should have access to good-quality healthcare that is centred on their needs and preferences [66].

Despite receiving verbal and/or written instructions for specimen self-collection, studies found that self-sampling interventions to diagnose STIs in women were feasible [7,51,60,61]. Similarly, participants who received verbal and written instructions for specimen self-collection reported ease and comfort in collecting their own specimens at their convenience [23,27,33,38,42,51,52,54,56]. This further highlights the ease with which self-sampling for STIs can be used as an alternative to clinic based STI healthcare management services. Based on these findings, the usefulness of self-sampling for STIs in resource-limited settings across the globe cannot be ignored. However, there is limited evidence of the uptake and adoption of such interventions in public STI healthcare-management services. Additionally, 63% of the included studies were conducted in HICs, and only 37% of the studies were conducted in LMICs. Similarly, Flowers et al. [63] reported increased uptake of self-sampling in the UK. The lack of evidence on the uptake of such interventions in LMICs is concerning. Much effort is still required from relevant stakeholders to fulfil goal 3.3 of the Sustainable Development Goals 2030 which aims to end epidemics of various communicable diseases [64].

Only two of the reviewed studies reported on the use of pooled samples to diagnose STIs and highlighted a gap in the use of pooled specimens. Pooling of specimens from the genital tract and extragenital tract has proven successful in detecting infections in individuals who practice oral and anal sex [62]. The lack of evidence on the use of pooled specimens for diagnosing STI is concerning in cases of anal and oral sex which may contribute to the spread of STI-causing pathogens to areas beyond the genital tract [67].

We reviewed studies reporting on the accuracy of diagnostic results when using self-sampled specimens. We found that self-sampled specimens result in fairly accurate diagnoses [50,68]. Self-sampled vaginal swabs, in particular, yielded similar results to physician-collected specimens [69,70]. The overall findings of the review highlighted that the diagnostic results on self-collected specimens were fairly accurate.

When considering high global STI statistics [1] and limited access to good-quality healthcare and laboratory services in LMIC [71,72], this lack of rapid POC testing is concerning. By providing services closer to patients, POC testing has the potential to improve the turn-around time for the management and treatment of disease which will improve disease outcomes [71,73].

Although STIs have been of great interest among the medical population, the level of public knowledge of such is not well known. It has been proven that sufficient knowledge about STIs has an effect on minimizing the spread of infection [74]. A study conducted in Italy about knowledge of STIs among young individuals reported that they had insufficient knowledge [75]. In South Africa knowledge about STIs was relatively good among women of childbearing age but there were gaps in knowledge [76]. Another study in Ethiopia reported low levels of good knowledge of STIs [77]. This highlights the need to make more efforts to educate individuals across the globe among different population age groups.

### 4.1. Strengths and Limitations

We conducted extensive searches on various databases and websites to retrieve all relevant studies. We used the PRISMA guidelines to guide the recording and reporting of our results thereby ensuring transparency. We did not have any language restrictions or study design limitations. We systematically identified relevant studies and charted and analysed data. Although we made every attempt to ensure a rigorous search strategy, we may have missed relevant studies. Our screening tool may not have been rigorous enough, resulting in the inclusion of 44 studies.

### 4.2. Implications for Practice

Most of the studies included in this review were conducted in HICs where there is equitable access to good-quality healthcare services. In HICs, the use of advanced innovative healthcare practices is normal. Few studies on self-sampling interventions were conducted in LMICs where access to good-quality healthcare services still poses a challenge for ordinary citizens. In LMICs, healthcare systems are far behind in terms of the services they provide to their people. As such, LMICs continue to struggle with health issues that are no longer a burden in HICs. Our review highlights the ease and usefulness of self-sampled vaginal swabs, which may prove feasible and adaptable in LIMCs.

The coronavirus disease of 2019 (COVID-19) pandemic has led to the minimization of human interaction and movement to reduce and prevent the spread of COVID-19.

According to Pinto et al. [78], COVID-19 restrictions do not only affect the way people interact with each other, but also the way humans interact with healthcare and STI management services. Thus, adding to the previously stated restrictions already posed by clinic based STI healthcare services. Furthermore, it is well known that COVID-19 restrictions also increased the acceptability of home-based healthcare services to ensure that patients continue to receive relevant healthcare services. As such, the use of self-sampling interventions to diagnose STIs would play an integral role as alternatives to clinic-based STI healthcare management services while observing COVID-19 restrictions and regulations. When considering the current burden of STIs in sub-Saharan Africa, the convenience of self-sampling during the COVID-19 pandemic, and the potential to improve STI management in this region, cannot be disregarded.

Despite the potential benefit of self-sampling in LMICs, we found no evidence for self-sampling interventions that had been developed according to the needs and preferences of women. There is a need to develop self-sampling interventions for STI diagnosis which are tailored to the preferences of the user.

### 4.3. Recommendations for Future Research

We found that most of the research on self-sampling for STIs was conducted in HICs. We recommend that future studies be conducted in LMICs. Self-sampling seems to largely rely on self-collected vaginal swabs and there is opportunity to investigate different types of self-sampling including tampons, sanitary pads, and urine, which may promote the development of a self-sampling intervention tailored to the preferences of women. Since only two studies reported on the use of pooled samples for diagnosing STI, we recommend future research investigating the use of pooled specimens to diagnose STIs present in extragenital areas. We also found that self-sampling and POC testing was rare in primary healthcare practice. Future research should explore the use of POC tests and self-sampling to bring healthcare services closer to users who have limited access to healthcare.

## 5. Conclusions

This scoping review shows that despite self-sampling interventions having the potential to improve STI management and treatment here is a need for self-sampling interventions tailored to the needs of users. Self-sampled vaginal swabs have the potential to increase access to healthcare. In LMIC settings, having women collect their own samples in private settings may save time and resources in primary care settings.

## Figures and Tables

**Table 1:** PCC framework for defining eligibility of studies to address the research question.

Criteria	Determinants	Description
Population	Women	Women of sexual reproductive age
Concept	Self-sampling interventions	Women collecting their own specimens for STI diagnosis, either at home or at a healthcare facility without the aid of a healthcare professional. The self-sampling specimen collection kit. Submission of self-collected specimens for diagnosis to a healthcare facility or directly to the laboratory. Feedback on patient results. Laboratory diagnostic techniques used for different specimen collection kits.
Context	STIs	STIs in women excluding Human Immunodeficiency Virus (HIV).

**Table 2:** Results of the database search.

Date	Database	Keywords	Number of Results Retrieved
14 July 2021	Scopus	(TITLE-ABS-KEY (sampling OR sample OR “self sampling” OR “self sample” OR “sti testing” OR “sti diagnosis” OR “sexually transmitted infections test*” OR “self-collect*” OR “sexually transmitted disease testing*”) AND TITLE-ABS-KEY (“Specimen Handling”) AND TITLE-ABS-KEY (“Sexually Transmitted Disease*” OR “sexually transmitted infection*”) AND TITLE-ABS-KEY (wom*n OR female* OR girl*) AND NOT TITLE-ABS-KEY (aids OR “HIV Infections” OR hiv OR “human immunodeficiency virus” OR “acquired immunodeficiency syndrome”))	117
15 July 2022	Cochrane	(sampling OR sample OR “self sampling” OR “self sample” OR “sti testing” OR “sti diagnosis” OR “sexually transmitted infections test*” OR “self-collect*” OR “sexually transmitted disease testing*”):ti,ab,kw (Word variations have been searched)	26
19 July 2021	PubMed	(((sampling[tw] OR sample[tw] OR “self sampling”[tw] OR “self sample”[tw] OR “sti testing”[tw] OR “sti diagnosis”[tw] OR “sexually transmitted infections test*”[tw] OR “self-collect*”[tw] OR “sexually transmitted disease testing*”[tw] AND (female[Filter])) AND (“Specimen Handling/methods”[Mesh] OR “Specimen Handling”[tw] AND (female[Filter]))) AND (“Sexually Transmitted Diseases, Bacterial”[Mesh] OR “Sexually Transmitted Diseases, Viral”[Mesh] OR “sexually transmitted infection*”[tw] OR “sexually transmitted disease*”[tw])) NOT (“HIV Infections”[Mesh] OR “HIV Infections”[tw])	213
19 July 2022	Web of Science	((((ALL=(sampling OR sample OR “self sampling” OR “self sample” OR “sti testing” OR “sti diagnosis” OR “sexually transmitted infections test*” OR “self-collect*” OR “sexually transmitted disease testing*”)) AND ALL=(“Sexually Transmitted Disease*” OR “sexually transmitted infection*” OR STI OR STD)) AND ALL=(wom*n OR female* OR girl*)) AND ALL=(“Specimen Handling” or “Specimen Collection” OR Specimen)) NOT ALL=(aids OR “HIV Infections” OR hiv OR “human immunodeficiency virus” OR “acquired immunodeficiency syndrome”)	311
21 July 2022	Medline (EBSCO)	(((ALL=(sampl* OR “self sampl*” OR “sti test*” OR “sti diagnosis” OR “sexually transmitted infections test*” OR “self-collect*” OR “sexually transmitted disease test*”))) AND ALL=( ) NOT ALL=(″)	140

**Table 3:** Summary of articles included in this scoping review on self-sampling interventions for diagnosing STIs in women.

Author	Country	Aim	Population and Sample Size	Self-Sampling Intervention	Diagnostic Test	Key Findings
Weisenfeld et al., 1996 [55]	USA	Agreement between physician-collected specimens and self-sampling in patients with urogenital CT.	*n* = 300 of which 200 self-samples and 100 samples from a pilot study	Vaginal introitus swab	Amplicor CT test	Vaginal introitus swabs, provider-collected to detect urogenital CT: sensitivity = 92% (95% coefficient of variation (CI), 83 to 100). Sensitivity of vaginal introitus swabs was greater than PCR, culture or enzyme immunoassay of the cervix or urethra. Self-sampling, PCR: sensitivity = 81%. Urine samples, PCR: sensitivity = 73%.
Ostergaard et al., 1996 [24]	Denmark	Self-sampling to collect urogenital samples at home, mailed to the laboratory for CT deoxyribonucleic acid (DNA) analysis. Diagnostic efficacy was compared to provider- collected urethral and endocervical swabs.	*n* = 222 aged 18–25 years	First-catch urine (FCU), vaginal pipette wash	Amplicor PCR	Prevalence of CT = 11.2% (23/205 women). Self-sampling, PCR: Sensitivity = 96%, specificity = 92.9%. Self-sampling, LCR: Sensitivity = 100%, specificity = 99.5%. Provider-collected: Sensitivity = 91%, specificity = 100%.
Tanaka et al., 2000 [31]	Japan	Compare vaginal swabs obtained by providers and self-sampling to screen for CT infection.	Group 1 = 193 men, 187 women Group 2 = 91 high-risk sex workers	Vaginal swab, FCU, endocervical sample	New generation amplified immunoassay IDEIA PCE chlamydia kit and PCR	Male urine samples and female endocervical swabs: IDEIA PCE performed similarly to the Amplicor PCR. Relative sensitivity of IDEIA (79.3%), IDEIA PCE (91.4%), and Amplicor PCR (100%) on male first-void urine specimens. Relative sensitivities of IDEIA (85%), IDEIA PCE (95%), and Amplicor PCR (100%) on female endocervical specimens. Self-sampled vaginal swabs (SVS), IDEIA PCE: positivity rate = 25.2%. Clinician-collected vaginal specimens, IDEIA PCE: positivity rate = 23.1%. Clinician-collected endocervical swabs, PCR and IDEIA PCE, positivity rate = 27.5%.
Tabrizi et al., 2000 [32]	Australia	Evaluate two commercial amplification systems detecting CT and NG from tampon specimens	*n* = 400 tampon specimens	Tampon specimens	In-house PCR assay, Abbott LCR, Roche cobas^®^ Amplicor	Detection of CT, commercial assays similar to in-house PCR (*p* = 0.68, *p* = 0.73). Detection of NG, in-house PCR superior to Abbott LCR (*p* = 0.0001) but similar to Roche PCR (*p* = 0.11). Roche PCR and LCR similar detection of CT. LCR testing of extracted DNA did not increase sensitivity.
Domeika et al., 2000 [48]	Lithuania	Using self-sampled and mailed specimens to detect genital CT	*n* = 94	Vaginal introital sample	PCR (AMPLICOR CT, Roche Diagnostic Systems, Inc., Branchburg, N)	CT, self-sampling, PCR vs. cell culture: Sensitivity = 100% Vaginal samples, PCR: Sensitivity = 100%, >PCR and cell culture on cervical samples. Single vaginal sampling, PCR: Sensitivity = 100%. Self-samples, mailed vaginal specimens are feasible for PCR-testing for genital CT. Self-sampling would help to reach a section of the population in which pelvic examination and cervical sampling are not routinely performed.
Macmillan et al., 2000 [38]	UK	The feasibility of using self-sampled vulval swabs, instead of FCU to diagnose female genital CT infection in a family planning population.	*n* = 103 younger than 25 years old	vulval swab, urine	LCR	Prevalence of CT = 11.7%. Vulval swabs had 100% sensitivity, 100% specificity, and 100% Positive Predictive Value (PPV) and Negative Predictive Value (NPV). FCU had 91.7% sensitivity, 100% specificity, and PPV = 100% and NPV = 98.9%. Women found both tests to be acceptable.
Rompalo et al., 2001 [35]	USA	Evaluate a single intra- vaginal swab (SIS) for simultaneous detection of NG, CT, Trachomatis vaginalis (TV), and HPV infections among military women on active duty.	*n* = 793	Intravaginal swab (a Dacron SIS from the AMPLICOR collection kit)	A combination test that uses PCR combined with DNA probe hybridization in a colorimetric detection assay.	NG culture: sensitivity = 70.8%, specificity = 100%. NG PCR: sensitivity = 95.8%, specificity = 97.8%. CT enzyme immunoassay: sensitivity = 72.8%, specificity = 90%. CT PCR: sensitivity = 94.6%, specificity = 99.3%. Self-sampling with an SIS accurately detects multiple STIs.
Alary et al., 2001 [53]	Canada	Evaluate a modified sanitary napkin as a self-sampling device to detect CT infection in women. Self-sampled specimens vs. endocervical and FCU from the same women.	*n* = 246	Modified sanitary napkin, FCU	cobas^®^ Amplicor PCR	Modified sanitary napkin, PCR: sensitivity = 93.1% (95% CI, 83.3 to 98.1%), specificity = 98.9% (95% CI, 97.4 to 99.6%). FCU, PCR: sensitivity = 81.0% (95% CI, 68.6 to 90.1%), specificity = 100% (95% CI, 99.2 to 100%). Modified sanitary napkin: PPV = 91.5% (54 of 59), NPV = 99.1% (447 of 451). Urine samples: PPV = 100% (47 of 47), NPV = 97.6% (451 of 462). Modified sanitary napkins may be an effective non-invasive device for self- sampling to detect urogenital CT infection.
Harper et al., 2002 [20]	USA	Compare the detection of high-risk HPV using tampons with longer exposure times in the cervicovaginal vault vs. self-sampling swabs. Women’s acceptance of sampling with a tampon for longer periods.	*n* = 103 aged 16 years and older.	Tampon	PCR	309 tampons vs. 618 self-sampled swabs, 83% were returned. Among women, the 10-s tampon detected fewer with normal histology and high-risk HPV (HR-HPV) relative to swabs (*p* = 0.0412). The 1 h, 4 h, and overnight tampons had similar detection rates to swabs. In women with cervical intraepithelial neoplasma (CIN), tampons and swabs similarly identified HR-HPV.
Holland-Hall et al., 2002 [54]	USA	The use of self-sampling to screen female adolescent detainees for three organisms in a setting where speculum exams are not feasible.	Sample size not indicated	Vaginal swab	PCR	Self-sampling and endocervical testing yielded similar results for NG (K: 0.614, *p* = 0.001), CT (K: 0.865, *p* = 0.001). Self-sampling and vaginal microscopy yielded similar results for TV (K: 0.627, *p* = 0.001). All participants supported the practice of self-sampling using a vaginal swab. All participants stated willingness to perform self-testing in between their regular pelvic exams.
Knox et al., 2002 [41]	Australia	Compared FCU, SVS, self-sampled tampon and practitioner-collected endocervical swab specimens to detect NG, CT and TV.	*n* = 318	Vaginal swab, urine, tampon, endocervical swab	Culture, wet prep and Nucleic Acid Amplification Test (NAAT) PCR	Detection rate, PCR: CT = 11.5%, NG = 11.8%, TV = 24.6%. PCR significantly more sensitive than microscopy and culture in detecting NG and TV. CT, PCR: Sensitivity, tampons = 100%; FCU = 72.7% NG, PCR: Sensitivity, tampons = 97.2%, endocervical swab = 92.6%, self-sampled swab = 71.9%, FCU = 31.2%. Sensitivity of urine PCR for detecting NG improved with freezing of urine specimens and shorter transport time. TV, PCR: Sensitivity, tampons = 100%, TV = 87.7%.
Chandeying et al., 2003 [49]	Thailand	Compared several specimen types to detect CT infection. Assess the acceptability of self-sampling.	*n* = 953	urine, vaginal swab, tampon	PCR	CT prevalence = 17.6% amongst female sex workers (FSWs) and 5.7% amongst outpatient women. Acceptability: Tampo*n* = 72.6%, self-sampled vaginal swab = 74.2%. In FSWs: Sensitivity, tampo*n* = 95.9%, SVS = 89.2%, more sensitive than either urine or endocervical swabs. In outpatient women: Sensitivity, endocervical swabs = 100%, tampons and SVS = 85.7%. Specificity was >98% for all sampling methods for both groups.
Shafer et al., 2003 [33]	USA	Compare FCU, self-collected vaginal swabs and physician-collected endocervical specimens to detect CT and NG in a large cohort of young women upon entering the military.	*n* = 2157	FCU and vaginal swab	NAAT—LCR	SVS: best detection of CT and NG. CT, detection rate: FCU = 72%, endocervical specime*n* = 64%, FCU/vaginal swab = 94. Women preferred self-sampling to routine pelvic examinations.
Ogilvie et al., 2005 [61]	n/a	Meta-analysis comparing the accuracy of patient-collected vaginal specimens with clinician- collected specimens for detecting HPV-DNA.	*n* = 106 studies	Multiple specimen types, Dacron, cotton swab, cytobrush, tampons	PCR, Hybrid Capture II (HCII)	Self-sampling vs. clinician-collected specimens: sensitivity = 0.74, specificity = 0.88. Self-sampling in referral settings: sensitivity = 0.81, specificity = 0.90. Tampons offered sensitivity between 0.67–0.94 (*n* = 4 studies). PCR and HC-II offered similar sensitivity.
Karwalajtys et al., 2006 [57]	Canada	Agreement physician obtained cervical and SVS to detect HPV DNA. Women’s preferences for collection method according to age	*n* = 543 women aged 15 to 49 years and a group of 50 years and older	SVS	HC-II assay for carcinogenic HPV	*n* = 307 women, aged 15–49 years. Prevalence of HPV: vaginal swabs = 20.8% (64/307), cervical specimens = 17.6% (54/307). Prevalence of HPV, women older than 50 years, vaginal swabs = 9.9% (15/152), cervical specimens = 8.6% (13/152). Vaginal swabs vs. cervical specimens: Agreement ƙ = 0.54 (younger women) and ƙ = 0.37 (older women) (both *p* < 0.001), indicating fair agreement. Nearly half of women preferred self- sampling or had no preference.
Van de Wijgert et al., 2006 [27]	South Africa	Self-sampling using vaginal swabs or tampons compared to physician- obtained swabs	*n* = 450	Tampon, vaginal swab	cobas^®^ Amplicor CT/NG test, TV by MDM culture, bacterial vaginosis (BV) by Nugent scoring of a Gram-stain slide, 22 Candida species by Sabdex culture, and high-risk HPV types by the Digene HC-II for hrHPV DNA Test.	Self-sampling (tampons and swabs): satisfactory validity for NG, CT, BV, and Candida species. Self-sampling (swabs): satisfactory validity for HR-HPV. Self-sampling was not suitable for diagnosing TV by culture. Self-sampling was feasible and acceptable, but some women preferred speculum examinations, which allowed the clinician to view the vagina and cervix.
Morris and Rose 2007 [18]	Not indicated	HPV detection as primary cervical cancer screening	Sample size not indicated	Tampons, vaginal swabs	PCR NAAT	PCR tests for HPV show high test sensitivity and reliability PCR tests for HPV could be adopted as a stand-alone test, and, if positive, other tests such as p16INK4a or cytology could be used to increase specificity. Women can self-sample and send samples to laboratories Self-sampling is convenient and easy. Suited to the lifestyles and busy schedules of the modern woman.
Kucinskiene et al., 2007 [40]	Lithuania	The utility of self-sampling and pooling of samples for screening for CT among sexually active students.	*n* = 424	Vaginal swabs	Digene HC-II CT/NG Test	CT was present in 30 (5.6%) of 533 vaginal samples. Out of the 177 pools (three samples per pool), 29 pools were positive for CT/NG. 26 positive pools contained at least one positive CT sample and two contained two positive CT samples. The remaining CT/NG positive pool was only positive for NG. HC-II, pooled vaginal samples: Sensitivity = 100%, specificity = 100%. 30 (7.1%) sexually active students (20–24 years old, *n* = 424) tested positive for CT. Prevalence in high schools ranged from 0 to 1%. Prevalence in college students was as high as 14.2%.
Winer et al., 2007 [29]	USA	SVS vs. physician-collected cervical vs. physician-collected vulvovaginal swabs in women. Compared ability of mailed samples and in-clinic self-collected samples to detect HPV DNA.	*n* = 374	Vaginal swab	HPV PCR analysis	HPV detection: physician-collected cervical/vulvovaginal > clinician-collected vulvovaginal > self-sampled vaginal > clinician-collected cervical Agreement between sampling modalities: women (25 to 30 years) = 86.5–95.7% (κ 0.65–0.92); women (18 to 25 years) = 94.9–98.8% (κ 0.84–0.96).
Safaeian et al., 2007 [34]	Uganda	Compare SVS and physician-collected cervical swabs in their ability to detect HPV DNA.	*n* = 2223	Vaginal swab	HC-II determined carcinogenic HPV. PCR to determine HPV genotypes.	More than 86% of women complied with self-sampling, only 51% accepted a pelvic examination. HR-HPV, prevalence = 19% (self-sampling and physician-collected samples) Self-sampling vs. physician-collected sampling: agreement = 92% (κ = 0.75), HIV-positive (ƙ = 0.71), HIV-negative (ƙ = 0.75).
Fang et al., 2008 [45]	USA	Concordance of two self-sampling methods (FCU vs. vaginal swab) and provider-collected endocervical samples for detecting CT and NG	*n* = 350 aged 12–18 years	FCU and self-sampled vaginal swabs	BDProbeTec ET Amplified DNA Assay	*n* = 342 adolescents CT positivity rate = 26.6 per 100 women NG positivity rate = 11.7 per 100 women Vaginal swab: Sensitivity, CT = 97.3%, NG = 100% FCU: Sensitivity, CT = 89.2%, NG = 88.6% Provider-collected sample (PES): Sensitivity, CT = 90.1%, NG = 95.5% Specificities: 94.7%~99.7% for CT and NG. Agreement, CT: SVS vs. PES (ƙ = 0.89), SVS vs. FCU (ƙ = 0.88) and PES vs. FCU (ƙ = 0.91) (*p* < 0.0001) Agreement, NG: SVS vs. PES (ƙ = 0.91), SVS vs. FCU (ƙ = 0.87) and PES vs. FCU (ƙ = 0.91) (*p* < 0.0001).
Bialasiewicz et al., 2009 [58]	Australia	A novel, super-absorbent polymer-based method for self-collection and ambient temperature transport of urine. Evaluate ability to detect CT.	52 urine specimens	Urine	PCR for CT (cobas^®^ TaqMan 48 rtPCR)	Gel-based urine sample vs. neat urine: Sensitivity = 94.6–100%, specificity = 100% No PCR inhibition or reduced analytical sensitivity using gel-based samples.
Falk et al., 2010 [46]	Sweden	Sensitivity of self-sampled vaginal specimens, FCU, self-sampled specimens/FCU and endocervical specimens to detect genital CT in asymptomatic women.	*n* = 318	Vaginal swab, FCU, endocervical specimens	cobas^®^ Amplicor CT Test, LightMix 480HT PCR OLBIOL GmbH, (Berlin, Germany) on a LightCycler 480	172 of 318 women tested positive for CT. 19 (16.8%) of asymptomatic women (*n* = 113) had discordant tests (FCU vs. self-sampling) and 7 (12.1%) of symptomatic women (*n* = 58) had discordant tests (FCU vs. self-sampling). CT, sensitivity: endocervical specimens = 97.1% (166/171), self-sampled specimens = 96.5% (165/171) and self-sampled vaginal/FCU specimens = 95.3% (163/171), FCU = 87.7% (150/171), which was significantly lower.
van Dommelen et al., 2011 [59]	The Netherlands	Performance of SVS/FCU combination compared FCU or vaginal swabs alone.	*n* = 791	SVS, first-catch urine (FCU)	NAAT: Strand Displacement Amplification (SDA) assay and PCR	CT detection rate: SVS = 94% (89%–99%), FCU = 90% (84%–96%), SVS/FCU = 94% (89%–99%) (NAAT by SDA and PCR) Detection rates were similar across sample types. SVS vs. FCU, agreement = 98% (*p* = 0.61) SVS vs. SVS/FCU, agreement = 99% (*p* = 1) FCU vs. SVS/FCU, agreement = 98.8% (*p* = 0.51)
Stewart et al., 2012 [25]	UK	Accuracy of self-sampled vulvovaginal swabs vs. clinician-taken urethral and endocervical swabs for detecting NG in women attending a sexual health clinic in an urban setting	*n* = 3973 older than 16 years	Self-sampled vulvovaginal swab	NAAT— Aptima Combo 2 (AC2)	Culture: sensitivity = 81% Clinician taken endocervical NAATs: sensitivity = 96% Self-sampled vulvovaginal NAATs: sensitivity = 99% AC2 tests were significantly more sensitive than culture (*p* < 0.001). Endocervical vs. vulvovaginal swabs: No difference. Therefore, the specificities and PPV of all tests in all sites were 100%, and NPV of all tests were 99% or greater. Culture: sensitivity = 84%. Clinician-taken endocervical AC2: sensitivity = 100%. Self-sampled vulvovaginal swab AC2: sensitivity = 100%. AC2 assays were significantly more sensitive than culture (*p* = 0.004) for both endocervical and endocervical swabs.
Levy et al., 2012 [39]	Not indicated	Specimen collection and test characteristics of NAATs at different anatomical sites.	Sample size not indicated	Self-collection: urethra, cervicovaginal, rectum and pharynx.	NAATs	NG/CT detection: urine samples for men, self-sampled vaginal swabs in women.
Jang et al., 2012 [19]	Not indicated	Compare SVS and FCU to diagnose TV	*n* = 530	Dacron swab taken from an APTIMA collection kit, nylon-flocked swab, FCU	Transcription-mediated amplification analyte-specific reagents using a cutoff of 50 000 relative light units.	Only seven of 75 women infected with TV reported symptoms. Self-sampling: Sensitivity = 97.2%, specificity = 97.6% FCU: Sensitivity = 41.7%, specificity = 100%. Dacron swab: Sensitivity = 92.3%, specificity = 98.8%. Flocked-nylon swab: Sensitivity 92.3%, specificity = 99.2%.
Jones et al., 2013 [60]	Brazil, South Africa	Evaluated the XenoStrip TV test, now the OSOM Trichomonas rapid test in two developing countries. Compared home- and clinic-based screenings. The home arm required two self-sampled vaginal swabs.	Sample size not indicated, Women aged 14–25 in South Africa.Women aged 18 to 40 years in Brazil	SVS	PCR and rapid point-of-care test (POCT)	Specificity for self-testing using the rapid TV test was high in both settings. South Africa: sensitivity = 83.3%; Brazil: sensitivity = 68.4% (non-significant, z test *p* = 0.2). Pooled sensitivity = 76.7% (95% CI, 61.4 to 88.2%). Pooled specificity = 99.1% (95% CI, 98.2 to 99.6%). Self-sample, PCR: specificity = 99.1%, 95% CI, 98.2 to 99.6%), sensitivity = 76.7%; 95% CI, 61.4 to 88.2%). Sensitivity was higher among symptomatic women (87.5%; 95% CI, 47.3 to 99.7%) than asymptomatic women (80%; CI, 51.9 to 95.7%).
Geelen et al., 2013 [44]	Nether-lands	Clinical performance of rectal and self-sampled vaginal swabs for detecting of CT and NG	*n* = 921	Rectal swab, self-sampled vaginal swabs	Roche cobas^®^ 4800 CT/NG assay and Abbott m2000 real-time™ CT/NG	Rectal swabs: High concordance rates for detecting CT and NG ( ≥ 96%) using the cobas^®^ 4800 and the Abbot m2000 real-time™ assay. κ coefficients > 0.75, indicating excellent agreement. Self-sampled vaginal swabs: High concordance rate (≥99%) using the cobas^®^ 4800 and Abbot m2000 real-time™ assays for detecting CT and NG.
Ting et al., 2013 [30]	Kenya	Compare APTIMA HR-HPV mRNA testing of physician-collected and self-sampled specimens for detecting high-grade cervical lesions in high-risk FSWs in Kenya. Identify risk factors for HR-HPV mRNA in our population of FSWs	*n* = 350 aged 18 to 49 years	self-sampled specimen using the APTIMA Cervical Specimen Collection and Transport cytobrush	Aptima HPV (AHPV), AC2, Aptima TV (ATV)	Prevalence: hrHPV mRNA, physician collected samples = 30%, self-sampled specimens = 29%. Prevalence high-grade squamous intraepithelial lesion (HSIL) = 4% (*n* = 15). HSIL, HR-HPV testing: Sensitivity, physician-collected samples = 86% (95% CI, 62%–98%), self-sampled specimens = 79% (95% CI, 55–95%). HSIL, HR-HPV testing: Specificity, physician-collected samples = 73% (95% CI, 68%–79%), self-samples specimens = 75% (95% CI, 70%–79%). Risk factors for HPV: age < 30 years, TV or Mycoplasma genitalium (MG) infection, more than eight years of educational cbasattainment.
Van Der Pol et al., 2013 [28]	USA	Patient infection status derived from vaginal swab specimens compared with other sample types	*n* = 4279	FCU; a single vaginal swab, Self-collected or clinician-collected using the cobas^®^ collection kit	NAAT, cobas^®^ CT/NG (c4800) Test (Roche Diagnostics, Indianapolis, IN) performed on the cobas^®^ 4800 system	Detection rates: CT = 248, NG = 65 CT, self-collected vs. other samples, agreement = 98.8% to 99.2%, ƙ = 0.88 NG, Self-collected vs. other samples, agreement = 99.8% to 99,9%, ƙ = 0.92
Chernesky et al., 2014 [21]	Canada	Compared self-sampled cervical collection and transportation (SCT) samples to PreservCyt and SurePath cervical samples	*n* = 580	Self- collected vaginal sample using SCT	Aptima HPV assay, a target NAAT	Cervical SCT vs. PreservCyt samples: agreement = 91.1%; J = 0.82, AHPV assay Cervical SCT vs. SurePath samples: agreement = 86.7%; J = 0.72, AHPV assay. Self-sampled vaginal SCT vs. physician- collected SCT: agreement = 84.7%; J = 0.68, *p* = 0.014, 3.35 times more extra positives in self-sampled vaginal SCT Self-sampled vaginal SCT vs. cervical SCT samples: agreement = 82.0%; J = 0.63, *p* = 0.046, similar extra positives Women found the kit easy to use and comfortable for self-sampling
Li et al., 2014 [22]	USA	Compare AC2 performance on combinations of vaginal swabs, transportation media and FCU samples.	*n* = 287	flocked swab, Aptima vaginal swab, FCU	AC2	37/287 women tested positive for CT. All samples were detected by the Aptima swab, the flocked swab in the Aptima specimen transport medium and the ESwab in ESwab medium. Aptima swabs in Aptima specimen transport uniquely detected CT in three swabs. Flocked swabs in Aptima specimen transport medium uniquely detected CT in two swabs. CT, FCU: Sensitivity = 100%.
Chernesky et al., 2014 [56]	Canada	Compare a specimen collection and transport (SCT) kit for detecting CT and TV from SVS and physician-collected vaginal and cervical samples	*n* = 708	vaginal swab using the SCT kit Self-vaginal: S-VSCT Physician- collected vaginal (P-VSCT)Physician-collected cervical: (P-CSCT)	CT: AC2, TV: ATV	84.3% of women were comfortable with collecting specimens. 87.4% of women, 25 years and older, were comfortable with self-sampling 78.8% of women, younger than 25, were comfortable with self-sampling. CT, agreement: S-VSCT vs. P-VSCT 99.6% (ƙ = 0.93). S-VSCT vs. CSCT 99.4% (ƙ = 0.91), S-VSCT vs. PC L-Pap 99.4% (ƙ = 0.91), S-VSCT vs. P L-Pap 99.3% (ƙ = 0.88). TV, agreement: S-VSCT vs. P-VSCT 99.9% (ƙ = 0.97), S-VSCT vs. P-VSCT 99.7% (ƙ = 0.94), S-VSCT vs. PC L-Pap 99.6% (ƙ = 0.91), S-VSCT vs. SP L-Pap 98.8% (ƙ = 0.78).
Boggan et al., 2015 [51]	Haiti	Feasibility of HPV screening as primary testing for cervical cancer. Compare vaginal self-sampling to physician- administered cervical screening methods	*n* = 1845 aged between 25–65 years.	Vaginal swabs	HR-HPV genotyping using the HC-II HPV assay pool.	HR-HPV screening is a feasible tool for primary cervical cancer screening in a low-resource, Haitian population. Women volunteered to participate in vaginal self-screening for HPV. Sensitivity of HPV screening for detecting ≥CIN-II: vaginal samples = 87.5%, cervical samples = 96.9%. Cervical vs. vaginal samples: High agreement. Vaginal self-sampling sample can be implemented in this under-screened and high-risk population.
Arias et al., 2016 [52]	Canada	Survey opinions of young sexually active women on ease and comfort of self-sampling using HerSwab. Agreement between self-sampling and provider-collected swabs for detecting CT and NG.	*n* = 189 aged16–41 years	Vaginal swab collected with a HerSwab device	AC2	Respondents (97.1%) reported that the HerSwab instructions were easy to follow. 80.9% of respondents preferred self-collection over physician collection. 79.7% (137/172) of respondents would consider self-sampling at home. 96.2% (177/184) of respondents found it easy or very easy to insert and withdraw the device. 93.4% (171/183) of respondents found it easy and very easy to turn the device handle while inside the vagina. Agreement: self-sampling vs. provider collected specimen, CT: 94.7% (90.2%–97.3%; κ = 0.64 (0.43–0.85)) Agreement: self-sampling vs. provider- collected specimen, NG: 98.4% (95.1–99.6; κ = 0.56 [0.13–1]).
Obiri-Yeboah et al., 2017 [36]	Ghana	The performance of self-collected cervico-vaginal samples for detecting HPV compared to clinician collection	*n* = 333	vaginal swab using careHPV brush	careHPV assay	HPV: agreement between self-collected and clinician-collected samples = 94.2% (ƙ = 0.88, *p* ≤ 0.0001) HIV seropositive: agreement between self-collected and Clinicia-collected samples, ƙ = 0.84 (*p* < 0.0001) HIV seronegative: agreement between self-collected and clinician-collected samples, ƙ = 0.86 (*p* < 0.0001) self-collected vs. clinician-collected: sensitivity = 92.6% (95% CI: 85.3–97.0%), specificity = 95.9% (95% CI: 89.8– 98.9%).
de Marais et al., 2018 [47]	USA	Clinical performance of self-sampling cervico-vaginal specimens for detecting CIN-II in US women at risk of cervical cancer due to underscreening. Compare self-sampled specimens and physician-collected specimens to detect CT, NG, TV, and MG	*n* = 284	Cervico-vaginal swab, using Viba brush	AHPV, AC2 assay for CT and NG, the ATV assay and the Aptima analyte-specific reagent-based assay for MG	Detection rate: 193 of 284 women were at high risk for HPV, irrespective of sampling and cytology. Self-sampling: Detected high-risk HPV in all cases of HSIL and CIN-II + TV, detection: Self-sampling = 10.2%, Physicia*n* = 10.8% MG, detection: Self-sampling = 3.3%, Physicia*n* = 5.5% CT, detection: Self-sampling = 1.1%, Physicia*n* = 2.1% NG, detection: Self-sampling = 0%, Physicia*n* = 0.5%. High-risk HPV: Self-sampling ƙ = 0.56, Physician ƙ = 0.66 TV: Self-sampling ƙ = 0.86, Physician ƙ = 0.91. MG: Self-sampling ƙ = 0.65, Physician ƙ = 0.83. Most participants understood self-collection instructions (93.6%) and were willing to use self-collection in the future (96.3%).
Lockhart et al., 2018 [23]	Kenya	The agreement of SCT for CT, NG, TV and MG screening using self-versus physician-collected specimens. The acceptability of self-sampling for female sex workers (FSWs) over 18 months.	ages 18 to 49 years, sample size not indicated	self-sampled cervico-vaginal sample using the Aptima Cervical Specimen Collection and Transport cytobrush	CT, NG: the Aptima Combo 2 assay TV, MG: the ATV assay	Prevalence, SCT: NG = 2.9%, CT = 5.2%, TV = 9.2%, MG = 20.1%. Prevalence, physician-collected: NG = 2.3%, CT = 3.7%, TV = 7.2%, MG = 12.9%. Agreement between samples was consistently strong (ƙ range, 0.66–1.00) for all STIs, except for MG which had a moderate agreement (ƙ range, 0.50–0.75). Most participants found self-collection easy (94%) and comfortable (89%). SCT was effective for STI screening in a clinic-based, less-developed country setting.
Khan et al., 2019 [43]	India	Reliability of self-sampled vaginal swabs vs. physician-collected swabs to diagnose fungal (Candida albicans or non- albicans Candida species) bacterial vaginosis (BV) and parasitic TV aetiology of vaginal discharge and prevalence of various infections and coinfections.	*n* = 550	Vaginal swabs	Gram staining, wet mount, and culture	Prevalence: Bacterial vaginosis (*n* = 79, 14.4%), vulvovaginal candida (VVC) (*n* = 144, 26.2%) and TV (*n* = 3, 0.5%) VVC coexisted with BV in 58 (10.5%) patients. No coinfection of TV with BV or VVC. Candida albicans was isolated in 84 (58.3%) VVC cases. Self-sampling, BV: sensitivity = 91.1%, specificity = 100%, PPV = 100%, NPV = 98.5% Self-sampling, Candida albicans VVC and TV: sensitivity (100%), specificity (100%), PPV (100%) and NPV (100%). Self-sampling vs. physician, agreement: ƙ = 0.95 (BV), ƙ = 0.99 (VVC), ƙ= 1.0 (TV).With specific instructions and guidance, self-collected swabs can approximate physician-collected swabs.
McLarty et al., 2019 [37]	USA	Compare tampons, self- sampled vaginal swabs and physician- collected specimens to diagnose HPV.	*n* = 174	Tampons, swabs (Eve Medical HerSwab)	Roche cobas^®^ HPV method	HR-HPV prevalence = 13.5% (*n* = 174) All physician-collected specimens were sufficient for detecting HPV. 15 (27%) of tampon specimens were of poor quality. 1 (2%) of vaginal swabs were of poor quality. Vaginal swabs were similar to physician-collected specimens, while tampons were of poor quality.
Nodjikouambaye et al., 2019 [6]	Chad	Performance of a novel genital veil (V-Veil-Up Gyn Collection Device, V-Veil-Up Pharma Ltd., Nicosia, Cyprus) for self- sampling to diagnose STIs as compared to physician- collected specimens.	*n* = 271	Self-sampling with veil	IVD-marked multiplex real-time PCR Allplex STI Essential Assay	Genital mycoplasmas detected in 54.2% of samples. Ureasplasma parvum detected in 42.6% of samples. Self-sampling performed similarly to physician-collected samples in detecting genital microorganisms. Sensitivity = 97% (95%CI: 92.5–99.2%), specificity = 88.0% (95%CI: 80.7–93.3%).
Verougstra et al., 2020 [26]	Belgium	The feasibility of molecular testing for CT and NG in pooled versus single site samples in a large cohort of FSWs.	*n* = 501	a pharyngeal swab, a self-collected vaginal swab and a self-collected rectal swab	NAAT using Abbott Real Time	*n* = 489 patients, prevalence: CT = 6.5% (95% CI 4.5% to 9.1%), NG = 3.5% (95% CI 2.0% to 5.5%), CT and NG coinfections = 1.4% 42 patients tested positive on at least one non-pooled sample. Only five tested negative in the pooled sample. CT: Sensitivity = 94% (95% CI 79% to 99%). NG: Sensitivity = 82% (95% CI 57% to 96%). Missed pooled samples derived from single-site infections with low bacterial loads. Testing only vaginal samples would have missed 40% of CT infections and 60% of NG infections.
Kim et al., 2021 [42]	Korea	Do self-sampled vaginal specimens contain enough DNA to detect HPV. Compare self-sampled specimens with physician-collected cervical samples. Investigated ease, comfort and reliability of a self-sampling to obtain a vaginal sample.	*n* = 151	vaginal swab—(using G+ Kit^®^; DocTool)	PCR: the Anyplex II HPV28 Detection assay, Real-time PCR using CFX96.	Prevalence HPV, PCR: self-sampling = 67.5%, physician-collected = 57.4%. Prevalence, high-risk (HR) HPV, PCR: self-sampling = 58.7%, physician-collected = 48.6% Sensitivity, HR HPV: self-sampling = 100% (95% CI 0.09 to 0.32) for high-grade squamous intraepithelial lesion, 78% (95% CI –0.09 to 0.13) for atypical squamous cells, 95% (95% CI –0.01 to 0.25) for low-grade squamous intraepithelial lesion. Self-sampled specimens contained enough DNA to detect HPV. Self-sampled vs. physician-collected samples had similar sensitivity and specificity. Self-sampling is feasible for detecting abnormal cervical cytology. Self-sampling is easy and reliable.

## Data Availability

The data for the scoping review was obtained through secondary data analysis and as such the original datasets were not presented. All data supporting the conclusions of this scoping review are available through the reference list.

## Abbreviations

AC2	Aptima Combo 2
ATV	Aptima Trichomonas vaginalis
AHPV	Aptima Human Papilloma Virus
BV	Bacterial vaginosis
CT	*Chlamydia trachomatis*
CI	Confidence interval
CIN-II	Cervical intraepithelial lesion
FSW	Female sex workers
FCU	First-catch urine
HC-II	Hybrid Capture II
HSIL	High-grade squamous intraepithelial lesion
HR-HPV	High-risk human papilloma virus
HIC	High-Income Country
HPV	Human Papilloma Virus
LMIC	Low- and middle-income countries
LCR	Ligase chain reaction
MG	Mycoplasma genitalium
mPCR/RLB	Multiplex polymerase chain reaction/reverse line blot
NG	*Neisseria gonorrhoea*
NPV	Negative predictive value
NAAT	Nucleic Acid Amplification Test
P-VSCT	Physician-collected specimen collection and transport
POCT	Point-of-care testing
PPV	Positive predictive value
POC	Point-of-Care
PCR	Polymerase Chain Reaction
PC-VS	Patient-collected vaginal swab
PRISMA-ScR	Preferred Reporting Items for Systematic Reviews and Meta-analyses extension for Scoping Reviews
PRISMA	Preferred Reporting Items for Systematic Reviews and Meta-analyses
STI	Sexually Transmitted infections
SCT	Self-collection and transport
S-VSCT	Self-collected specimen collection and transport
SIS	Single intravaginal swab
SVS	Self-collected vaginal swab
TV	Trichomonas vaginalis
USA	United States of America
UK	United Kingdom
VVC	Vulvovaginal Candida
